# CD8 and CD4 T Cells in West Nile Virus Immunity and Pathogenesis

**DOI:** 10.3390/v5102573

**Published:** 2013-10-22

**Authors:** Jason Netland, Michael J. Bevan

**Affiliations:** 1 Department of Immunology, University of Washington, Seattle, WA 98109, USA; E-Mail: jnetland@uw.edu; 2 The Howard Hughes Medical Institute, University of Washington, Seattle, WA 98109, USA

**Keywords:** West Nile Virus, T cells, CD8 T cells, CD4 T cells

## Abstract

CD4 and CD8 T lymphocytes are adaptive immune cells that play a key role in the immune response to pathogens. They have been extensively studied in a variety of model systems and the mechanisms by which they function are well described. However, the responses by these cell types vary widely from pathogen to pathogen. In this review, we will discuss the role of CD8 and CD4 T cells in the immune response to West Nile virus infection.

## 1. Introduction

West Nile Virus (WNV) is a single-stranded positive sense RNA virus of the Flaviviridae family. It is endemic in regions of Africa, Asia, The Middle East and Europe, spread to the western hemisphere in the mid 1990s and was first identified in the United States in 1999 [1,2]. The virus is maintained in bird and mosquito reservoirs with humans and other mammals as incidental hosts. Infection in humans results in a wide range of disease severity. In most individuals, infection is controlled and no disease is evident. In about 20% of cases however, infection causes systemic febrile illness and in a subset of these individuals virus spreads to the central nervous system causing meningitis, encephalitis, or acute flaccid paralysis syndrome [3]. 

The factors determining disease severity are not entirely known, although the immune system is known to play an important role. The elderly and those with impaired immune systems are at greater risk to develop severe neurological disease [4,5]. This observation has been substantiated in animal studies where it has been shown that numerous components of the immune systems, including inflammatory cytokines and chemokines, complement, B cells and T cells are important in controlling viral replication and limiting disease [6]. This review will focus on the role of CD4 and CD8 T cells in WNV infection.

## 2. CD8 T Cells

Early studies demonstrated the importance of CD8 T cells in protecting against WNV infection. Rag-deficient mice, which lack all T cells and B cells, are highly susceptible to infection, and while passive antibody transfer was able to limit acute disease, virus eventually returned to cause severe disease and death, suggesting a role for T cells in clearing virus [7]. This notion was confirmed when CD8 T cells were transferred into Rag-deficient hosts and were able to rescue the majority of the mice from lethal disease [8]. Additionally, mice deficient in CD8 or class Ia major histocompatibity complex (MHC-I) exhibited much greater mortality than wild-type mice [9]. The kinetics of viral replication at early time points in these knockout strains was unaltered, but viral titers remained high in the spleen after day 8 when it had been cleared from the wildtype. Additionally, virus replicated to a greater extent in the brains of knockout mice and persisted in surviving mice for greater than 30 days post-infection. Thus CD8 T cells play an important role in clearing WNV and limiting disease severity, particularly in the CNS. 

In contrast, studies have described a potentially pathogenic role for CD8 T cells in WNV infection [10,11]. When CD8^−/−^ mice were infected with a low dose of the Sarafend strain of WNV they exhibited increased mortality. Conversely, when a high dose of virus was administered, CD8 deficiency resulted in increased survival [11]. Virus and T cells were present in the brains much earlier post-infection following high dose infection, which could result in increased neuronal death as CD8 T cells exert their effector functions, leading to the increase in mortality. Additionally, when mice lacking the interferon stimulated gene Ifit1 were treated with CD8-depleting antibody, they survived an average of 3 days longer, indicating CD8 T cells contribute to mortality [11]. CD8 T cells have been suggested to play a pathogenic role in a number of diseases, particularly in the CNS [12,13,14]. Therefore, while CD8 T cells appear to be required for control of WNV infection, they may contribute to disease in some situations.

CD8 T cells control viral infection via several mechanisms including direct cytotoxicity using perforin, granzyme, TRAIL or Fas-FasL interactions, or through the secretion of antiviral cytokines such as tumor necrosis factor (TNF) and gamma interferon (IFNγ) [15,16,17]. The mechanisms CD8 T cells use to control WNV have been extensively studied using genetic knockout of various effector molecules. The ability of CD8 T cells to directly kill virally infected cells is a vital element in their control of WNV as mice lacking perforin, FasL or TRAIL all exhibited increased mortality following infection [18,19,20,21]. In all cases, the kinetics of viral replication in the periphery was normal, but increased viral burdens were detected in the CNS. As was observed with CD8 and MHC-I knock-out mice, virus was found to persist long term in the CNS of all cytotoxic effector molecule knock-out mice, further supporting the notion that CD8 T cells are particularly important in clearing virus from the CNS. Adoptive transfer of wild-type CD8 T cells but not those lacking the various effector functions was able to limit CNS infection and mortality in CD8^−/−^ [18,19,20,21] or Rag^−/−^ [22] mice, showing that these molecules are important specifically in CD8 T cells. 

Gamma interferon (IFNγ) has been demonstrated to have a protective role in numerous viral infections [23,24,25,26,27,28] and is produced by CD8 T cells in response to WNV infection in both mice [29] and humans [30]. However, the role for IFNγ production by CD8 T cells is unclear. In one study, when IFNγ-deficient T cells were transferred into Rag^−/−^ hosts, they provided little to no protection while IFNγ-sufficient cells protected [22]. Alternatively, another group showed adoptive transfer of IFNγ-deficient T cells protected mice from lethal infection as well as wild-type cells [10]. It should be noted that the latter study was conducted with the Sarafend strain of WNV that does not cause increased disease severity in IFNγ^−/−^ mice. This is in contrast to infections with the lineage I New York strain (WNV-NY), which results in increased susceptibility in the absence of IFNγ [31,32]. While the authors contended that the loss of IFNγ production from γ/δ T cells is responsible for the phenotype, the contribution of IFNγ from CD8 T cells has not been directly tested.

One of the strongest correlates of severe disease in humans is advanced age [4,5]. This has also been observed in mice [22]. While aging has been shown to alter many aspects of both the innate and adaptive immune system, T cell defects are often the most severe [33,34]. Therefore, the effect of aging on CD8 T cells responding to WNV infection has been examined in both mice and humans. Aged mice display an impaired CD8 T cell response, in terms of both the number of antigen-specific cells and their functionality [22]. Furthermore, adoptive transfer experiments demonstrated a marked defect in the ability of T cells from aged mice to protect from lethal disease. Similar results were obtained when mice were vaccinated with a single-cycle virus particle vaccine, with aged mice generating smaller primary and memory T cells responses compared to adult mice [35]. Interestingly, when these mice were challenge with WNV, the recall responses in aged mice were equal to or exceeded those observed in adults. Thus, defects in CD8 T cell function appear to play a role in the enhanced disease associated with aging, but aged mice are still able to produce a robust response with multiple stimulations.

In humans the situation is less clear with no evidence of CD8 T cell defects correlating with aging. In a sample of 40 infected patients, the CD8 T cell responses were the same or increased in the aged cohort compared to younger individuals [36]. Furthermore, while more memory phenotype cells were observed in those with neuroinvasive disease, this phenotype did not correlate with age. Additional studies found similar results, with no differences in the number of CD8 T cells [30,37] or their ability to produce effector cytokines between aged and young cohorts [30]. Furthermore, the diversity of the CD8 response, indicated by the number of epitopes recognized, was not reduced in aged individuals [37]. Therefore, while WNV-specific CD8 T cells appear to be defective in aged mice, which may at least partially explain the increased morbidity and mortality observed, the same is not true in humans.

**Figure 1:**
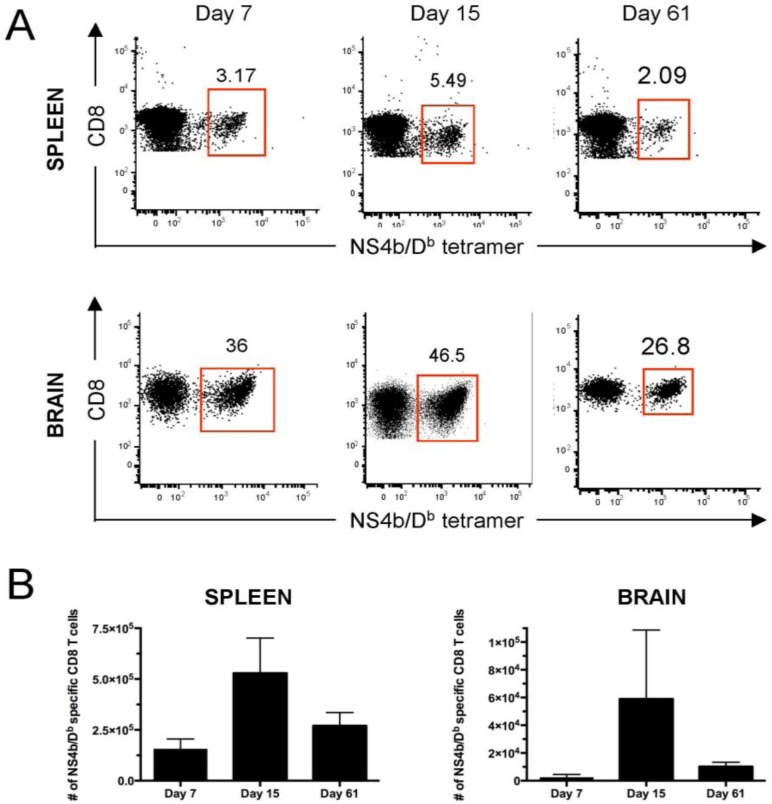
C57Bl/6 mice were infected subcutaneously with 1000 PFU WNV-TX. At the indicated time points post-infection spleen and brain were harvested and cells were isolated. T cells were stained with anti-CD8 antibody and MHC-I tetramer containing the immunodominant epitope within the NS4b protein (SSVWNATTA) and analyzed by flow cytometry. (**A**) Representative dot plots gated on CD8+ cells; (**B**) Total number of NS4b/D^b^+ cells from two experiments combined (n = 5–6).

Recently efforts have been made to determine the antigen specificity of CD8 T cells responding to WNV in both mice and humans. In mouse studies, one group used “peptide overload” and overlapping peptide pools to map one immunodominant and numerous subdominant epitopes [8]. They observed that the hierarchy of these responses was maintained in both effector and memory cells. By generating CTL clones specific to these epitopes and transferring them individually into Rag^−/−^ mice they demonstrated that T cells specific for the immunodominant and one subdominant epitope were able to protect the majority of mice from lethal disease while another subdominant epitope protected to a lesser extent. Another group employed a computational prediction approach to identify two immunodominant epitopes, both of which were also identified in the study above. They also generated CTL clones specific to these epitopes, which were able to lyse infected target cells *in vitro* and decrease mortality *in vivo*. CD8 T cells from C57BL/6 mice specific to the immunodominant NS4b epitope have been shown to be generated after infection in wild-type mice and can form stable memory populations [38] in both the spleen and brain (Figure 1).

A variety of methods have also been used to identify epitopes in humans. The use of bioinformatic prediction followed by testing against patient’s samples identified 26 novel epitopes restricted by 11 different class I HLA alleles [39]. Comparative mass spectroscopy has also been used to identify epitopes. In one study, six peptides were identified, with one being immunodominant, two being subdominant and three exhibiting little activity [40]. In a separate study, six different epitopes were discovered with one being immunodominant, three subdominant and two showing little activity [41]. In both studies, the epitopes were widely distributed among the WNV proteins, with epitopes identified in the envelope, capsid, NS2b, NS3, NS4b and NS5 proteins. These and future studies identifying viral epitopes and examining the function of epitope-specific cells will be useful in developing a vaccine against WNV.

While a vaccine against WNV is available for horses [42,43], no vaccine has been approved for human use. Most vaccination strategies against viruses focus on antibody neutralization, however given the importance of CD8 T cells in clearing viral reservoirs in WNV infection, an effective vaccine would likely need to elicit a strong CD8 T cell response. In one study, vaccination with single-chain HLA-A2 MHC trimers incorporating an immunodominant human epitope partially protected HLA-A2 transgenic mice from lethal disease [44]. The vaccine induced a strong CD8 T cell response and reduced viral titers in the brains. In a separate study, mice vaccinated with either inactivated virus or a DNA plasmid that produces virus-like particles had higher survival rates than those that received placebo [45]. A single dose of these vaccines induced both virus-specific antibody and CD8 T cells, and the importance of CD8 T cells was evident as their absence (either by antibody depletion or genetic deficiency) resulted in increased mortality. However, the induction of CD8 T cells was not absolutely required for vaccine-induced protection in these experiments as mice that received a booster dose of vaccine were protected in their absence. This is presumably due to the increased neutralizing antibody titers found in boosted animals being sufficient to clear the virus. Despite this, these studies clearly demonstrate a role for CD8 T cells in vaccine-induced protection from WNV and suggest that generation a robust T cell response should be an important component of future vaccines. 

## 3. CD4 T Cells

While the role of CD8 T cells in WNV has been fairly well described, the contribution of CD4 T cells is less well understood. Infection of mice that lack CD4 T cells either by antibody depletion or genetic deficiencies in CD4 or class II MHC results in enhanced disease and mortality [45]. While the kinetics of viral replication were unaltered when assayed in the spleen and serum, increased virus was detected in the CNS and remained high for at least 45 days post-infection and all mice succumbed to infection by day 50. Serum antibody levels and CD8 T cell infiltration into the CNS was unaltered at early time points, but both were impaired in the absence of CD4 T cells at late time points, suggesting CD4 T cells contribute to control of WNV by providing help to B cells and CD8 T cells at late stages of infection. Supporting this notion, activated CD4 T cells have been detected in the CNS for at least 12 weeks following WNV infection [38]. 

Some evidence suggests CD4 T cells may play a direct role in limiting WNV replication. As described above, Rag^−/−^ mice (which lack both B and T cells) are highly susceptible to WNV infection. When naïve CD4 T cells were adoptively transferred into Rag^−/−^ mice, WNV induced mortality was reduced from 97% to 20% [46]. Furthermore, after identifying CD4 T cell epitopes, peptide vaccination of immunocompetent mice resulted in increased survival. Epitope-specific CD4 T cells were shown to produce IFNγ, IL-2 and granzyme B and directly lysed target cells both *in vitro* and *in vivo*. Indirect evidence for a direct role of CD4 T cells in controlling WNV infection comes from the study of mice lacking IL-1R1 [47]. These mice exhibit increased susceptibility and increased virus in the CNS. While numbers of both CD8 and CD4 T cells were increased in the knockout mice, the CD4 T cells were impaired in their ability to elicit effector function (IFNγ production) upon restimulation, while CD8 T cells responded normally. Thus, CD4 T cells can contribute to the control of WNV in the absence of CD8 T cells and B cells, presumably by inflammatory cytokine production and directly targeting infected cells.

Regulatory T cells (T_R_) are a subset of CD4 T cells that can suppress effector CD4 and CD8 T cells and have been shown to play an important role in a variety of viral infections [48]. In humans infected with WNV, T_R_ cells were found to be expanded in the blood, and those patients exhibiting symptoms had lower T_R_ frequencies than asymptomatic individuals [49]. Infected mice displayed a somewhat similar phenotype with decreased frequency of T_R_ cells in symptomatic mice but frequencies did not increase after infection as was seen in humans. However, others have reported T_R_ expansion after WNV infection in mice [50]. When T_R_ cells were depleted, mortality significantly increased [49]. These observations suggest that T_R_ cells may play a role in limiting WNV disease, perhaps by limiting pathogenic aspects of the immune response, or by controlling the tempo of the response and the migration of effector cells as has been observed in other infections [51,52,53,54]. Further studies are necessary to determine the mechanisms of T_R_ protection in WNV infected humans and mice.

## 4. Innate Immune Signaling and T Cells

Numerous recent studies have revealed the importance of innate immune signaling molecules in shaping the T cell response to West Nile virus in both a cell extrinsic and intrinsic manner. Type I interferon (IFN) has long been appreciated as an essential factor in controlling WNV infection [6,55] and is known to play a role in shaping T cell responses to other viral infections [56,57]. To assess the role of type I IFN on T cell at various stages of infection, Pinto *et al.* utilized a blocking antibody against IFN-αβ receptor (IFNAR) [58]. They found that treatment prior to infection resulted in greatly increased numbers of virus specific CD8 T cells but blockade at day 4 post-infection did not alter CD8 T cell numbers. However, blockade at day 4 resulted in defects in T cell function as these cells produced less IFNγ, TNF and granzyme B and had increased expression of the exhaustion markers PD-1 and CTLA-4. This phenotype was not due to changes in T_R_ cells as there were no difference in the frequency or total number of these cells between untreated and treated animals. Adoptive transfer experiments revealed the effect of IFNAR signaling on T cells to be non-cell intrinsic. Thus, in addition to its role in controlling early viral replication, Type I IFN plays an important role in shaping the CD8 T cell response shortly after their initial priming.

IL-1 is another proinflammatory cytokine that has recently been demonstrated to play a role in generating an effective T cell response to WNV. When IL-1 receptor (IL-1R) is knocked out, mice display a phenotype similar to that observed when T cells are knocked out, with intact virus control in the periphery but impaired control in the CNS and subsequent mortality [47,59]. One study observed reduced quality of CD8 effectors indicted by reduced frequency of TNF- and TNF/IFNγ double-producing cells in the CNS of IL-1R^−/−^ mice, with no difference in IFNγ single-producing cells [59]. Alternatively, another group reported defective CD4 effector function, but no differences in CD8 T cells [47]. This discrepancy is likely due to the fact that the later study only examined IFNγ and granzyme B production and not TNF. IL-1 appears to mediate its effect on T cells via CD11c+ dendritic cells as adoptive transfer of these cells into IL-1R^−/−^ mice restored T cell function in the CNS and decreased mortality [47].

Pattern recognition receptors (PRRs) play a key role in the detection of WNV and induction of innate immune mechanisms that limit viral replication at early stages of infection [6]. Recently, a role for these molecules in shaping the T cell response has been appreciated. Toll-like receptors were the first group of PRRs identified and several are known to be activated following infection with viruses, including WNV [60,61,62,63,64,65,66]. MyD88, a key signaling adaptor for many TLRs has been shown to be important in controlling WNV infection [67,68]. In addition to directly inhibiting viral replication, it was demonstrated to play are role in recruiting CD8 and CD4 T cells to the brain by inducing chemokine induction [67]. Consistent with this, TLR7, which signals through MyD88, has also been shown to be necessary for effective control of WNV [68]. TLR7^−/−^ mice exhibited deficient leukocyte recruitment to the brain, likely due to reduced expression of the chemokines IL-12 and IL-23. 

RIG-I-like receptors (RLRs) are another family of PRRs that play a vital role in the recognition and control of WNV, mainly through the induction of type I IFN [6]. But like TLRs, recent evidence suggests RLRs and components of their signaling pathways contribute to protection by influencing the T cell response following infection. Genetic deficiency of MDA5, a RLR that detects dsRNA, results in increased viral burdens in the CNS and mortality. While there were no differences in the peripheral CD8 compartment, subtle phenotypic differences were observed in the CNS of MDA5^−/−^ mice. Adoptive transfer experiments confirmed that subtle defects in CD8 T cells resulted in defective viral clearance in the CNS and that this phenotype is non-cell-autonomous as MDA5^−/−^ CD8s primed in a MDA^+/+^ environment effectively cleared virus [69]. IPS-1, a signal adapator protein shared by MDA5 and RIG-I has also been shown to influence the T cell response to WNV [50]. Infection of IPS-1^−/−^ mice results in uncontrolled viral replication in numerous tissues and increased CD8 T cells in the CNS. T_R_ cells, which normally expand following WNV infection as discussed above, did not expand in the absence of IPS-1, which may contribute to the enhanced CD8 T cell response. Similarly, mice lacking IRF-1, a transcription factor downstream of PRRs, exhibit enhanced CD8 T cell proliferation in response to WNV infection [70]. Unlike with IPS-1^−/−^ mice, T_R_ expansion remained relatively intact, thus not likely explaining the enhanced expansion observed. Adoptive transfer studies revealed IRF-1 acts both within T cells and in their environment to influence proliferation. LGP2, a RLR who function in innate immune defenses is less well defined due to the fact that it lacks the CARD domain used by other RLRs to signal, has recently been shown to play a cell intrinsic role in CD8 T cell responding to WNV and lymphocytic choriomeningitis virus [71]. Mice lacking LGP2 exhibit reduced numbers of total and epitope-specific CD8 T cells in both the spleen and brain and this correlated with increased apoptosis of these cells at late timepoints. This survival defect may be due to increased sensitivity to CD95-mediated cell death as LGP2^−/−^ T cells were more susceptible to CD95L treatment *in vitro.* Thus, PRRs and their downstream signaling components play a key role in shaping the T cell response to WNV via a variety of mechanism.

## 5. Conclusions

In summary, CD4 and CD8 T cells have been shown to play a vital role in host defense against West Nile virus. In particular, these cell types appear to play a key role in clearing virus, especially in the brain. Since neuroinvasion results in the most severe disease and can result in death, understanding the mechanisms by which T cells limit or prevent virus replication in the brain is key. Additionally, with the recent identification of both CD4 and CD8 epitopes from WNV, future studies investigating the characteristics and functions of these epitope-specific cells will be instrumental to our understanding how the immune systems responds to WNV and in the development of vaccines to prevent severe disease from the infection.
